# Visual spatial location influences selection of instinctive behaviours in mouse

**DOI:** 10.1098/rsos.230034

**Published:** 2023-04-26

**Authors:** Samuel G. Solomon, Hadrien Janbon, Adam Bimson, Thomas Wheatcroft

**Affiliations:** Institute of Behavioural Neuroscience and Department of Experimental Psychology, University College London, London WC1H 0AP, UK

**Keywords:** visual field, approach, freeze, escape, rodent

## Abstract

Visual stimuli can elicit instinctive approach and avoidance behaviours. In mouse, vision is known to be important for both avoidance of an overhead threat and approach toward a potential terrestrial prey. The stimuli used to characterize these behaviours, however, vary in both spatial location (overhead or near the ground plane) and visual feature (rapidly expanding disc or slowly moving disc). We therefore asked how mice responded to the same visual features presented in each location. We found that a looming black disc induced escape behaviour when presented overhead or to the side of the animal, but the escapes produced by side-looms were less vigorous and often preceded by freezing behaviour. Similarly, small moving discs induced freezing behaviour when presented overhead or to the side of the animal, but side sweeps also elicited approach behaviours, such that mice explored the area of the arena near where the stimulus had been presented. Our observations therefore show that mice combine cues to the location and features of visual stimuli when selecting among potential behaviours.

## 1. Introduction

Vision helps animals to find food and avoid danger. Visually guided behaviours are therefore often key to survival, even in animals where vision does not seem to be the primary sense organ. For example, recent work has established that mice, while lacking high visual acuity, use vision to hunt prey including crickets [1,2], and often spontaneously approach a small dark disc (emulating a prey) presented near the ground plane via a computer monitor [3]. Visual stimuli can also elicit instinctive defence behaviours: mice escape to refuge when a rapidly expanding dark disc (loom) is presented on a computer monitor placed above the animal [4].

How mice respond to a visual stimulus depends on the features of that stimulus. When an overhead stimulus is a small moving dark disc, mice usually freeze rather than escape [5], and mice show little response to a bright overhead-loom stimulus [4]. Similarly, mice are more likely to approach slowly moving terrestrial objects and less likely to approach faster ones [3]. The influence of other visual cues is not known, but the fact that a small dark disc can elicit approach behaviour when it is near the ground plane [3], and defensive freeze behaviour when it is above the animal [5], suggests that the selection of behaviour also depends on the spatial location of a visual stimulus. If true, the asymmetry between behavioural responses to aerial and terrestrial objects may be ecologically adaptive—aerial objects are more likely to be threats, while terrestrial objects may be threats or prey. Indeed, it remains unclear which, if any, terrestrial visual stimuli elicit defence behaviours in rodents. Limited reports suggest that mice can also freeze if a small moving disc is presented near the ground plane [3], but they appear to show no response to a loom stimulus presented from the side [6]. Mice also show little or no response to a loom stimulus presented on a computer monitor directly below the animal [4,6]. Similarly, rats escape from a moving bar presented overhead, but not when it is presented to the side [7].

Comparison of behavioural responses across studies is made difficult by the fact that the visually guided behaviour expressed by an individual mouse depends on factors that remain poorly understood. When exposed to an overhead-loom stimulus, group-housed animals are less likely to escape than socially isolated animals [8]; animals usually freeze instead of run when a refuge is unavailable [9,10]; and some cohorts of mice always escape, but others show mixture of freezing and escape [4]. Similarly, work that shows approach toward a small dark disc near the ground [1–3] has been conducted in arenas lacking a refuge, but it is not clear if presence of refuge is important for freeze behaviours [5,11]. We therefore sought to establish the impact of stimulus location and feature on visually guided instinctive behaviours in mice, by measuring responses to aerial and terrestrial stimuli under the same conditions.

## 2. Materials and methods

### 2.1. Animals and housing

Adult mice (C57BL/6, age 8–14 weeks; 3 female, 17 male; obtained from Charles River Laboratories at 6–7 weeks of age) were housed under an inverted 12 : 12 light/dark cycle, and provided with environmental enrichment (bedding material to allow foraging, cardboard tunnels and Aspen blocks) and ad libitum food and water. Omitting female animals did not alter the results, so data from all animals were used. All testing took place during the dark phase of the daily cycle. Animals were group housed in single-sex cages, except for two male mice, who were singly housed. Animals were habituated to handling for 5 days before behavioural measurements commenced. Each animal participated in one behavioural testing session per day for 3–5 consecutive days. The environment was cleaned between sessions. Ten animals were used to establish responses to ‘loom’ stimuli, and another 10 animals were used to establish responses to ‘sweep’ stimuli.

### 2.2. Behavioural arena

The experimental arena was a box (48 × 48 cm) with white plastic covering the walls (30 cm high); the floor of the arena was a white plastic sheet. An 8 × 8 cm hole was made in one of the arena's walls, allowing access to a dark, opaque shelter (20 cm wide, 10 cm deep, 10 cm high). One gamma-corrected, calibrated monitor (ProLite E1980SD Iiyama; Japan, screen size 30 × 38 cm) was suspended 30 cm above the floor and centred on the arena. The long edge of the monitor was parallel to the wall that allowed access to the refuge. The wall opposite the nest was replaced by another calibrated monitor of the same model, such that the arena floor met the monitor just above its bottom bezel; white plastic strips bridged small gaps between screen edges and adjacent arena walls. Monitors were held at the mean luminance (92 cd m^−2^). Infrared LEDs placed above the arena provided infrared illumination and a camera (Blackfly S Mono, Clear View Imaging, sampling rate 60 Hz) equipped with a 0.4× wide-angle lens and infrared filter was positioned above the side monitor.

### 2.3. Visual stimuli

Visual stimuli were produced by a Dell Precision Tower 3420 computer running Bonsai v. 2.5 [12] with BonVision package v. 0.1 [13]. The ‘loom’ stimulus consisted of five repetitions of the following sequence: a 1 cm diameter black disc appeared and its diameter then linearly increased to 25.5 cm over 250 ms; the disc then remained on the screen, at that size, for an additional 380 ms, and then disappeared for 500 ms. We note that in previous experiments we presented only one repetition of a loom stimulus [5]; we chose a repetitive sequence here to provide more certainty that we would detect a defence behaviour if present. The ‘sweep’ stimulus was a 2.5 cm black disk that appeared at one side of the monitor and then translated smoothly to the opposite side (38 cm) over 3.5 s. For a mouse in the centre of the arena, this would correspond to a visual speed of 20.5 degrees s^−1^ when overhead, and 25.5 degrees s^−1^ when to the side. For overhead and side stimulus presentations, a visual stimulus was triggered manually when the animal was approximately under the centre of the overhead monitor and was not facing the refuge. The stimulus generator specified stimulus onset by applying a brief pulse to an infrared LED surmounting the arena via an Arduino Uno. In the first experimental session, animals were allowed to habituate to the arena for 15 min; in subsequent sessions, habituation was 5 min. Four stimulus presentations (trials) were then attempted; the first stimulus was always presented on the side monitor, and we then interleaved presentations on overhead and side monitors. Behavioural testing finished when these stimulus presentations were completed, or 20 min had elapsed from end of the habituation period, whichever was shorter.

### 2.4. Video acquisition and analysis

Video frames were acquired continuously in the Bonsai environment. Animal position was extracted online using inbuilt Bonsai nodes (*BackgroundSubtraction*, *FindContours*, *BinaryRegionAnalysis*) and stored for further analysis. Transformation matrices were estimated from daily calibration images of a 9 × 9 chequerboard surface using the *cptform* function in Matlab (Mathworks, NA, Release 2020b). Online estimates of animal centroid were projected from image space to arena space using the *tforminv* function, filtered with a moving average filter of width five frames (0.083 s) and used to estimate animal position and speed. Escape was classified when speed exceeded 40 cm s^−1^ and animals subsequently reached the nest within 2 s. Freezing was classified as epochs in which speed fell below 2 cm s^−1^ and subsequently remained below that level for at least 0.5 s. Latencies of these behavioural responses were defined as time to reach a speed of 40 cm s^−1^ (escape) or fall below 2 cm s^−1^ (freeze). Baseline measurements were obtained by analysing initial 5 min of the daily habituation periods; 5 s video sequences were triggered when mice were in the centre 50% of the arena for at least 0.2 s and moving at less than 20 cm s^−1^, with a minimum of 1 s between sequence starts.

### 2.5. Statistical analyses

Estimates of movement speed were obtained for trials of each stimulus, or baseline measurements, and accumulated into separate matrices, with each row representing a single trial in a single mouse. To calculate probability of freeze response, we assessed the criteria for freeze (described above) at each time point in each trial and entered a 1 in the relevant bins if the criteria were met, and 0 otherwise. To estimate cumulative probability of freeze response, we found first occurrence of freeze response in each trial (if present), set all subsequent values for that trial to 1, then averaged across all trials. Parallel analyses yielded time series of cumulative probability of escape behaviour, or proximity to side monitor or nest. We performed X^2^ tests to compare behaviour in different stimulus conditions (using the cumulative probability of each behaviour at the end of the trial) and non-parametric (Wilcoxon rank sum) tests to compare latency or duration of behavioural response, or running speed.

## 3. Results

**Figure 1.**
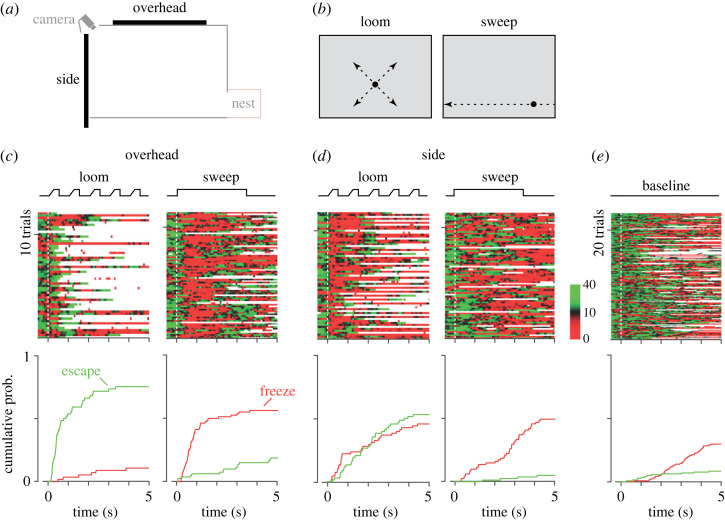
Dependence of freeze and flight behaviours on location in visual field. (*a*) Schematic of the experimental arena, as seen from the side. The arena was a white box, 48 cm wide and 30 cm high, with an aperture in one wall providing access to a refuge (nest). One monitor (overhead) was suspended above the arena, 30 cm above the floor. The other monitor (side) replaced the wall opposite the nest. (*b*) Schematics of the visual stimuli, as presented on the side monitor. The loom stimulus consisted of five sequential presentations of a black disc, that expanded from a diameter of 1 to 25.5 cm in 250 ms, persisted for 380 ms and then disappeared for 500 ms. The expansion point of the loom was 12.5 cm above the arena floor. The sweep stimulus was a 2.5 cm diameter black disc translating across the monitor over 3.5 s. The centre of the disc was 5 cm above the arena floor. The same stimuli were presented on the overhead monitor, except that they were centred on the screen. (*c–e*) Top: images showing the natural logarithm of movement speed in each trial (one trial per row), across all mice and all trials. Movement speed (in cm s^−1^) is indicated by the colourbar in (*d*): red indicates low speed; green indicates high speed; black indicates speeds close to the mean across animals; white indicates times when the animal was in the refuge. The time course of stimulus presentation is indicated by the lines above. Bottom: cumulative probability of having observed a flight (green) or freeze (red) response over time. (*c*) Behaviour during presentation of stimuli on the overhead monitor. (*d*) Behaviour during presentation of stimuli on the side monitor. (*e*) Behaviour during the absence of visual stimulus. Epochs were selected from the initial 5 min of habituation before each session, and subjected to the same analyses as in (*c*,*d*).

We placed naïve laboratory mice in an arena formed by a white box and monitored their movements with an infrared video camera (figure 1*a*). A small opening in one wall allowed animals to leave the arena and access a dark refuge (nest). Visual stimuli were presented either on a computer monitor placed over the centre of the arena (overhead), or on a ‘side’ monitor that replaced the wall opposite the nest.

### 3.1. Mice produce different patterns of defence behaviour for overhead- and side-looms

We first measured behavioural responses to a rapidly expanding black disc, emulating the looming silhouette of an approaching threat (figure 1*b*). To ensure that a behavioural response could be detected if present, this loom stimulus was repeated five times. We found that mice rapidly escaped to the refuge when the loom stimulus was presented on the overhead monitor (42/56 trials, 75%; response latency µ_1/2_ 0.45 s; peak running speed µ_1/2_ 71 cm s^−1^; figure 1*c*). We found freezing behaviour (epochs when movement speed fell below 2 cm s^−1^ for at least 0.5 s) in few trials (6/56, 11%). These observations are consistent with previous reports [4,5].

We interleaved trials of this overhead-loom stimulus with trials in which a loom stimulus was instead presented on the side monitor (figure 1*a,b*). We observed fewer escapes for side-looms (36/68 trials; 53%; figure 1*d*) than overhead-looms (X^2^ (1, *N* = 124) = 6.40 *p* = 0.011). The escapes induced by side-looms were also delayed (latency µ_1/2_ 1.79 s; *p* < 0.001 versus overhead, Wilcoxon rank sum) and slower (peak running speed µ_1/2_ 58 cm s^−1^; *p* < 0.001 versus overhead) than escapes induced by overhead-looms. Inspection of figure 1*d* suggests that many delayed escapes were preceded by an initial reduction in movement. Indeed, unlike for overhead-looms, side-looms frequently induced freezing behaviour (31/68 trials, 46%, met standard criteria for freezing; latency to start of freeze: µ_1/2_ 1.09 s). We note that we did not find a difference in overall probability of defence behaviours for side- and overhead-looms: we found either freeze or escape behaviour in 46/56 (82%) of overhead-loom trials, and in 53/68 (78%) of side-loom trials (X^2^ (1, *N* = 124) = 0.34, *p* = 0.561). Thus, while both overhead- and side-looms produce defence responses, overhead-looms usually produce escape to refuge, but side-looms produce escape and freezing behaviours.

### 3.2. Mice freeze during overhead sweeps and approach side sweeps

We previously showed that mice choose to freeze in response to more distal overhead threats, such as a small black disc slowly translating across the overhead field (‘sweeping’; [5]). We therefore measured responses to these sweeping discs, in a separate group of animals. We note that the speed of this stimulus on the retina will depend on the precise location of the animal within the arena: for a mouse in the centre of the arena, the sweeping stimulus would have a visual speed of 20.5 degrees s^−1^ when overhead, and 25.5 degrees s^−1^ when to the side. We found that mice rapidly engaged freezing behaviour when a sweep stimulus was presented overhead (45/80 trials, 56%; latency µ_1/2_ 0.65 s; figure 1*c*), and sometimes escaped to refuge (15/80 trials, 19%), in agreement with previous work. We found different behavioural responses when a sweep stimulus was presented on the side monitor, 5 cm above the arena floor (figure 1*d*). Freezing responses did occur during side sweeps (40/81 trials, 49%), and the duration of freezing was similar for overhead and side sweeps (µ_1/2_ 1.0 s in both cases). However, onset of freezing was not clearly time locked to the onset of a side sweep visual stimulus (latency µ_1/2_ 2.73 s; *p* < 0.001 versus overhead, Wilcoxon rank sum), and average movement speed during freezing was slightly greater during side sweeps (µ_1/2_ 0.68 cm s^−1^) than overhead sweeps (µ_1/2_ 0.54; *p* < 0.001). We also saw few escape responses (4/81 trials, 5%; X^2^ (1, *N* = 161) = 7.38, *p* = 0.006 versus overhead). Overall, we detected freezing or escape responses in 43/81 (53%) of side sweep trials, compared with 55/80 (69%) of overhead sweep trials (X^2^ (1, *N* = 161) = 4.15, *p* = 0.042). We conclude that side sweeps produce fewer escape responses, and weaker or delayed freezing responses, compared with overhead sweeps.

**Figure 2.**
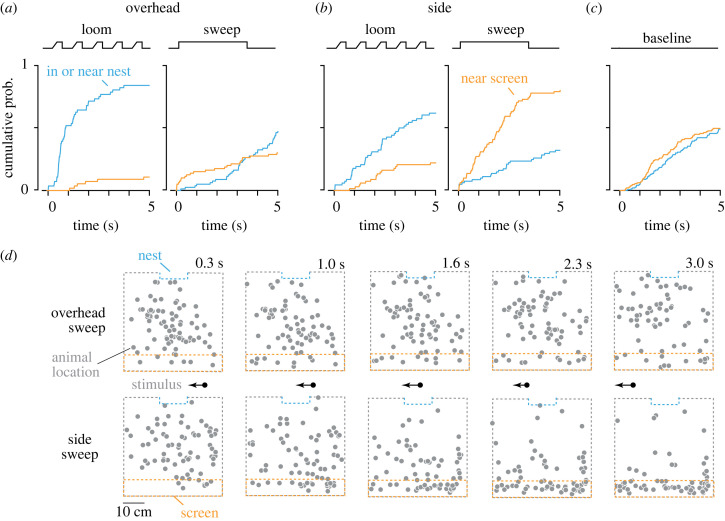
Small sweeping stimuli near the ground plane can induce approach behaviours. (*a–c*) Cumulative probability of the animal moving to within 8 cm of the side monitor (orange) or within 3 cm of the nest (blue), over time. Other conventions as in figure 1*c–e*. (*d*) Position snapshots of the animal position within the arena (as seen from above) during the presentation of an overhead sweep (top) or side sweep (bottom). Snapshots are provided for five time-points (0.3, 1, 1.6, 2.3 and 3 s) following the initial appearance of the sweep stimulus at the edge of the monitor. Each snapshot indicates the positions of animals at that time point, pooled across all trials, sessions and animals. The approximate position of the sweep stimulus on the monitor is indicated between the two series of snapshots.

Previous work, in absence of a refuge, showed that mice sometimes approach a small moving stimulus presented close to the ground plane [3]. We therefore asked whether our side sweeps, which apparently only weakly induce defence behaviours, induced approach behaviours. We tracked when mice moved to within 8 cm of the side screen and, for comparison, when they moved to within 3 cm of the nest (including times when they were in the nest). We first established how mice behaved when exploring the arena in absence of a visual stimulus (baseline). To do this, we analysed the first 5 min of each session and identified 5 s epochs triggered on when the mouse moved through the centre of the arena. In the absence of a visual stimulus, mice approached both the screen (50% of epochs) and nest (49%) (figure 2*c*).

We then applied the same analyses to behaviour during presentation of overhead and side sweeps. During or after presentation of an overhead sweep (figure 2*a,d*), mice investigated the screen (30% of trials) and nest (47%), not significantly different to baseline behaviour (X^2^ (1, *N* = 207) = 2.36, *p* = 0.124 versus baseline). By contrast, mice showed strong preference for the screen during presentation of side sweeps, approaching the screen in 80% of trials and the nest in 32% (figure 2*b,d*; X^2^ (1, *N* = 236) = 10.24, *p* = 0.001 versus baseline). Inspection of position snapshots (figure 2*d*) suggests that mice approached the screen and then investigated a limited area of it. They sometimes appeared to follow the moving target, but inconsistently, at least under these conditions (data not shown). Similar analyses applied to behaviour during presentation of overhead- and side-looms showed strong preference for the nest (84% and 62% of trials respectively), with little time spent near the screen (11%, 22%), as expected (figure 2*a,b*).

## 4. Discussion

We found that side stimuli, like overhead stimuli, can elicit defensive behaviours in mice. However, while side-looms often produced escapes, these were less vigorous than escapes produced by overhead-looms and were often preceded by freezing behaviour. Similarly, while animals showed epochs of immobility during presentation of side sweeps, these behavioural responses were less consistent than the freezing that could be produced by overhead sweeps. Side sweeps also elicited approach behaviours, such that mice explored an area of the arena near where the stimulus had been presented. To our knowledge, our observations provide the first evidence that visual stimuli do not need to be in the overhead visual field to elicit escape responses. By enabling direct comparison of behavioural response to overhead and side stimuli, our observations also show that how mice respond to visual stimuli depends on both spatial location and visual feature.

Some of our observations confirm previous work, made in absence of a refuge, which reported that small moving objects near the ground plane can elicit both immobility and approach behaviours in mice (e.g. [3]). Whether the immobility during presentation of a side sweep should be classified as ‘freezing’ is unclear, as there is no additional evidence that mice consider the side sweep a threat, so immobility in this context may be better thought of as helping prey detection or surveillance (see also [14]). More refined measures, such as postural changes [15] or cardiovascular changes [16], may be needed to establish whether epochs of immobility should all be classified as freezing. Similarly, while previous work has found no sex difference in responses to side sweep [3] or overhead-loom stimuli [4,17,18], most of our experiments were conducted on male mice, and future work might reveal sex differences.

We are aware of only one other study of behavioural response to side-looms in mice [6], which presented a series of multiple, relatively small loom stimuli in rapid succession (every 0.37 s); those stimuli elicited escapes when presented overhead, but not when presented to the side (freezing was not characterized). The slower and less vigorous escapes we observed for side-looms suggest that threat-response mechanisms are less sensitive to stimuli placed near the ground plane; we speculate that the stimuli used by [6] were too weak to induce them.

The mouse retina shows some topographic organization [19,20]. For example, ventral retina (overhead visual field) is poorly sampled by middle-wavelength (green) cone opsins and is therefore relatively more sensitive to UV light [21,22]. Similarly, some types of retinal ganglion cell may have higher resolution for objects in the overhead field (ventral retina) [19,20,23]. We found that both overhead- and side-looms produced rapid defence behaviours, so the retinal machinery needed to detect these stimuli must be present in both parts of the visual field. Topographic asymmetries may, however, lead to different neural representations of overhead and side stimuli, and thereby influence behavioural choices. For example, many or even most retinal ganglion cells will respond to a loom stimulus [24], and these generic neural responses may be sufficient to drive defence behaviours regardless of stimulus location. Faster and more reliable escape behaviours seen for overhead-looms may require the presence of specialized loom detectors [25]; if these detectors were biased to the overhead field, that may explain the behavioural differences. Better understanding of how behaviour depends on retinal stimulus position is likely to require fine-grained monitoring of eye and head position [7,26].

The different behaviours induced by overhead and side stimuli may instead be explained if the two parts of the visual field have different pathways through central brain areas. For example, the superior colliculus (SC) is important in instinctive behaviours (e.g. [16,17,27,28]). The superficial layers of SC are the major target of retinal axons in mouse [29], and these layers show strong topographic organization, such that the overhead visual field is represented in more medial parts of the SC, and the lower visual field is represented in more lateral parts. There is little evidence for mediolateral asymmetries in the organization of superficial layers of SC, and we found that freezing behaviours could be elicited by appropriate overhead or side stimuli. Freezing behaviours appear to be supported by pathways that course through optic layer of SC [28,30,31], part of the superficial layers, with projections that include pulvinar (also called the lateral posterior nucleus) and thence amygdala [10,32].

The deeper layers of SC, which receive direct projections from narrow-field neurons in superficial layers [33], are likely to be important in escape and capture behaviours. For example, ablation or suppression of narrow-field neurons in superficial SC reduces the probability of escape behaviours to overhead-looms, unveiling a freezing response [34], and reduces ability of mice to target prey during capture [27]. There are strong mediolateral asymmetries in deeper layers of SC that may explain why overhead objects more reliably trigger escapes, while some terrestrial objects appear to trigger approach. Medial parts of deeper layers are more likely to be connected to brain areas important in escape behaviours, while lateral parts are more likely to be connected to brain areas important in approach behaviours (see [17,18,28,35,36]). Consistently, the activation of narrow-field neurons in medial superficial SC elicits reliable, fast escapes while activation of the same neurons in lateral superficial SC elicits only occasional, slower escapes [34].

## 5. Conclusion

Our observations show that mice instinctively combine cues to the location and features of visual stimuli when selecting among potential behaviours. The different behavioural responses may be adaptive: looming aerial objects are more likely to be threats, while small terrestrial objects are more likely to be prey. But they also raise challenges for mouse survival (a looming terrestrial object also provides potential threat, but escape responses are slow) and it therefore seems likely that mice combine vision with other sensory modalities, such as audition, olfaction or somatosensation, to facilitate the detection of terrestrial threats (e.g. [37]). Regardless, the very different behaviours that can be elicited by different stimuli in the same location, or by the same stimulus in different locations, is likely to be useful in understanding how mice analyse and interpret sensory information to guide behaviour.

## Data Availability

Matlab tables containing tracking data and metadata, along with the Matlab script used for figure generation and analysis, are currently uploaded at Figshare. The digital link is https://figshare.com/s/dd319b2201249b42b7b7 [38].
